# Child and caregiver experiences and perceptions of asthma self-management

**DOI:** 10.1038/s41533-021-00253-9

**Published:** 2021-09-09

**Authors:** Lauren Kelada, Charlotte J. Molloy, Peter Hibbert, Louise K. Wiles, Claire Gardner, Emily Klineberg, Jeffrey Braithwaite, Adam Jaffe

**Affiliations:** 1 School of Women’s and Children’s Health, Faculty of Medicine and Health, UNSW Sydney, Sydney, NSW Australia; 2 Kids Cancer Centre, Sydney Children’s Hospital, Randwick, NSW Australia; 3 Australian Institute of Health Innovation, Macquarie University, Sydney, NSW Australia; 4 Australian Centre for Precision Health, Cancer Research Institute, School of Health Sciences, University of South Australia, Adelaide, SA Australia; 5 South Australian Health and Medical Research Institute, Adelaide, SA Australia; 6 Caring Futures Institute, College of Nursing and Health Sciences, Flinders University, Adelaide, SA Australia; 7 Ministry of Health, NSW Health, St Leonards, NSW Australia; 8 Respiratory Department, Sydney Children’s Hospital, Randwick, NSW Australia; 9 Aiming for Asthma Improvement in Children, Sydney Children’s Hospital, Randwick, NSW Australia

**Keywords:** Paediatric research, Asthma, Patient education

## Abstract

Asthma is the most common chronic condition of childhood. Self-management is integral to good asthma control. This qualitative paper explores how children with asthma and their parents perceive asthma, their experience with asthma, and how they manage symptoms, preventions and medications within and outside the home. We undertook 15 focus groups with 41 school-aged (6–11 years) children with asthma and 38 parents. Parents and their children attended the same focus groups. We used thematic analysis to analyse the transcripts. Our findings show the impact asthma can have on children’s social and emotional wellbeing and highlight how reliant school-aged children are on their parents to effectively manage their asthma. Parents reported being unsure when their child’s symptoms warranted visiting their doctor or hospital. Schools were identified as a source of difficulty regarding asthma management; families reported that children may be self-conscious about their asthma and using their inhaler at school. School policies and teachers’ lack of asthma knowledge were reported to exacerbate children’s reluctance to use their inhaler at school. Our results have implications for the design and implementation of children’s self-management interventions for their asthma, particularly when they are at school and away from their parents.

## Introduction

Asthma is the most common chronic condition of childhood with approximately 14% of children worldwide experiencing asthma symptoms^1^. In Australia, 1 in 10 children under the age of 15 years has asthma, with the highest prevalence in those aged 5–9 years (13%)^2^. Poorly controlled asthma is commonly observed^3^, related to poorer quality of life among children, and can impose significant burden on families and the health-care system^4^. Children with asthma access health-care services more frequently (416 hospitalisations per 100,000^5^), experience increased school absenteeism^6^, have sleep disturbance^7^ and restriction to everyday activity compared to children without asthma^8^. Caregivers of children with asthma also experience lower quality of life and higher workplace absenteeism than caregivers of children without asthma^6^.

Self-management is integral to good asthma control. Asthma clinical guidelines in Australia and internationally advocate for the inclusion of routine self-management education for patients with asthma^9–12^. Self-management programmes for children with asthma have important health benefits, including improved lung function, decreased morbidity, fewer days absent from school and reduced visits to emergency departments^13,14^. Emerging evidence also shows that apps and other digital media may be effective tools to facilitate asthma self-management, particularly among adolescents and adults^15–18^. However, further research is needed to assess the appropriateness and desired content of apps for children with asthma and their parents^19^.

Responsibility for management of a child’s asthma predominantly lies with the caregivers. However, it is important for children to begin to learn greater self-management as they commence school and spend increasing amounts of time away from direct parental care^20–22^. This transfer of responsibility for asthma management is likely to align with Piaget’s theory of cognitive development. As children move from the preoperational stage (2–7 years) to the concrete operational stage (7–11 years) of development, they begin to develop logical thinking skills which are necessary for following an asthma management plan^23^. However, school-aged children still lack the abstract thinking and planning skills required for the complex series of decision-making involved in autonomous asthma self-management. Therefore, to understand asthma management among children of this age group, the perspectives of both children and parents should be included.

It is important to understand families’ lived experiences of asthma management, both within and outside the home, given their central role in reducing asthma symptoms, as well as the substantial burden of disease imposed by childhood asthma. The aim of this study was to explore the experiences and perspectives of asthma and current and desired asthma self-management strategies of both children and caregivers, to enable identification of potential areas for improvement^24^.

## Methods

The current study was part of a larger project to develop an app to help families manage child asthma. The current study focusses on data gathered from focus groups to explore the experiences and perspectives of children with asthma and their caregivers. The study design was qualitative. The consolidated criteria for reporting qualitative research (COREQ) checklist is shown in Supplementary File 1. This research protocol was approved by the University of New South Wales Human Research Ethics Committee (no: HC15733), with recruitment through schools approved by NSW Department of Education (no. 16/890151), Queensland Department of Education and Training (no: 550/27/1745), and South Australian Department of Education and Child Development (no: DECD CS/16/00066-1.4).

### Participants and recruitment

Recruitment of participants (children and their parents) occurred in 2017 across four Australian states: New South Wales (NSW), Queensland (QLD), South Australia (SA) and Victoria (VIC) via e-newsletter circulations, and online or physical noticeboard advertisements through Asthma Australia (the peak national consumer body), schools and Facebook. Inclusion criteria for children–caregiver pairs were children with a medical diagnosis of asthma, aged 6–11 years, proficiency in speaking and understanding English, and without behavioural or intellectual disabilities that might preclude their ability to participate in group discussions or use. Interested families contacted one of the researchers (C.G.) via email and were screened for eligibility. Families were then provided (via email) with a study information sheet and consent form. Signed informed consent was obtained from caregivers prior to partaking in the study, with oral consent obtained from children prior to beginning the focus groups. Participants received an AUD$50 gift voucher as reimbursement for their time at the end of their focus group session.

### Study procedures and data collection

Child–caregiver pairs were divided into two groups according to the child’s age—Group A: 6–8 years and Group B: 9–11 years. Research has shown that for children age groupings are a crucial factor in group dynamics and discussion, with a 1–2 year age difference optimal, due to substantial differences by age in style, ability, and level of comprehension^25^. Focus groups comprised no more than six children in the same age group and took place at metropolitan research institutions in participants’ state of residence; no one other than participants and researchers were present.

The focus group incorporated two sessions: an initial group discussion immediately followed by hands-on user testing of asthma self-management apps; each session lasted for a maximum of 60 min (120 min in total). All sessions were audiotaped and transcribed verbatim for later analysis. Field notes were made during and after each Focus Group, with researchers (C.G. and C.J.M.) reaching consensus on the main points and data saturation. This paper reports on the findings of the initial group discussion.

Group discussions were semi-structured and led by a professional focus group facilitator and at least one member of the research team (C.G. and/or C.J.M.) (all females). In total, two professional facilitators were involved in the focus groups (one per session) and each held training in psychology and over 7 years’ experience in social and qualitative research and conducting focus groups with children. One research team member, the professional facilitator, and children with asthma and their caregivers were present at each focus group.

The group discussions were split into four sections: (1) asthma perceptions and feelings; (2) current experience of self-managing asthma; (3) desired asthma self-management strategies; and (4) self-management technology and asthma. To maximally engage the children multiple discussion formats were utilised. Sections one and two were undertaken in a group discussion and small task format, with children and their caregivers together. In sections three and four, caregivers and children were split into separate groups. Caregivers continued in a group discussion format while children completed a drawing or collage activity to design an asthma management “machine” using the prompt “create a machine that takes you from your asthma feeling bad to feeling good”. Drawing and collage modalities were utilised because they can help depict thoughts that are difficult to communicate verbally, especially for children^26,27^. These machines were used to prompt discussion rather than being analysed independently.

Focus group question and guides were developed by the authors and facilitators from a literature review and discussions with colleagues with qualitative, behavioural and/or clinical research expertise (Supplementary File 3). We used interview schedules drawn from Brown et al.^28^, Laster et al.^29^ and Shaw et al.^30^ as our key guides. There were also two pilot sessions conducted as per the described methods, with refinements to the focus group guides following each session.

Demographic information was collected via an online caregiver survey, including age, gender, ethnicity (Aboriginal or Torres Strait Islander), asthma profile (including year of diagnosis, diagnosis health-care professional, asthma care plan, number of general practitioner (GP) consults/emergency department (ED) hospital visits/hospital admissions in last 12 months related to asthma, and app usage (general/health or medical/asthma)).

### Analysis

We used thematic analysis of the transcripts to identify meaningful themes from the focus groups. We followed Braun and Clarke’s^31^ six-phase method of thematic analysis, using an iterative approach to analysis. We used NVivo Version 12 to code the focus group data. The coding tree is provided in Supplementary File 2.

Two researchers (L.K. and C.G.) separately read each interview transcript twice and created codes to indicate meaningful passages. Inter-rater reliability (*К* = 0.86) between the coders was high^32^ and we resolved discrepancies through discussion. The codes were discussed with a third researcher (L.M.) while referring to the original focus groups to ensure accuracy. The first author (L.K.) organised and interpreted the codes into overarching themes which accurately and meaningfully captured the original data, noting convergence and divergence across transcripts. L.K. then reread the focus group transcripts and discussed the themes with CG to finalise the analysis. Children’s machine creations are presented with each theme to help reflect that theme in the children’s words. Quotations (with participant IDs) are presented in-text to represent the themes.

## Results

### Participants

Over March and April 2017, 41 children and 38 caregivers participated in the focus groups across four Australian states (New South Wales [NSW], Queensland [QLD], South Australia [SA] and Victoria [VIC]), and caregiver surveys were completed for 37 children (Table 1). Focus groups ranged from 1 to 6 children per session (M = 2.60; SD *=* 1.3). An additional 22 families (36.7%; caregiver and child pairs) who had expressed interest in the study but had not yet returned the consent form were lost to follow-up prior to attending the focus groups. Reasons provided included illness, childcare arrangements (for other siblings) and other commitments (e.g., work). Children participating in the study were aged between 6 and 11 years (M = 8.30, SD = 1.6) and 51% were females. All caregivers who participated in the focus groups were females.

**Table 1:** Focus group participation by state.

By state	Number of sessions	Number of children/caregiver pairing (%)
SA	4	18 (44)
NSW	4	11^a^ (27)
VIC	4	7 (17)
QLD	3	5^b^ (12)

^a^There were only nine parents involved in NSW as there were two instances where one caregiver was present for two children (who both met the inclusion criteria).

^b^There were only four parents involved in QLD as there was one instance where one caregiver was present for two children (who both met the inclusion criteria).

#### Asthma profile

According to the caregiver surveys, none of the participants were newly diagnosed or new to managing asthma, with over half of the participants (*n* = 21, 57%) having been diagnosed with asthma for at least 6 years (Table 2). Most children (54%) had their asthma diagnosed by a GP, and 95% had a written asthma action plan (Table 2). Regarding health service utilisation, over half of children (52%) had more than four GP consults for asthma in the past 12 months, 39% had at least one emergency department presentation for their asthma in the past 12 months and one in six (16%) had an inpatient admission for their asthma in the past 12 months. Approximately three-quarters of children (76%) had used mobile phone apps (with or without parental support); however, less than 1 in 10 had ever used health/medical apps (8%) or those that were asthma specific (5%).

**Table 2:** Asthma profile of participants (*n* = 37).

Years since diagnosis by age group	Number of participants (% of sample)
6–8 year olds	21 (100)
2–5 years since diagnosis	14 (67)
6+ years since diagnosis	7 (33)
9–11 year olds	16 (100)
2–5 years since diagnosis	2 (13)
6+ years since diagnosis	14 (88)
Child identifies as Aboriginal or Torres Strait Islander	1 (2)
Diagnosing practitioner	
GP	20 (54)
Respiratory specialist	6 (16)
Paediatrician	4 (11)
Physician (not specified)	4 (11)
Hospital inpatient or ED	3 (8)
Current Asthma Care Plan (Yes)	35 (95)
GP consults for Asthma in past 12 months	
0–3	18 (49)
4–7	8 (22)
8–11	6 (16)
12+	5 (14)
ED visits for asthma in past 12 months (*n* = 36)	
0	22 (61)
1–3	11 (31)
4+	3 (8)
Inpatient admission for asthma in past 12 months	
0	31 (84)
1–2	6 (16)
Technology use before participation in study	
Used mobile apps	28 (76)
Used health or medical apps	3 (8)
Used asthma apps before	2 (5)

*GP* general practitioner, *ED* emergency department.

### Themes

We identified three themes from the data: (1) fear, sadness and frustration associated with asthma; (2) parental responsibility for proactively monitoring triggers, symptoms and medication; and (3) managing asthma at school requiring child communication about symptoms.

#### Fear, sadness and frustration associated with asthma

Children used positive adjectives to describe how they felt in the absence of their asthma symptoms. Children reported feeling happy, calm, excited, playful and energetic. When they were experiencing asthma symptoms, children reported feeling frustrated and sad, predominately due to missing out on opportunities to play with their friends (Box 1, Quote 1). One child described feeling frustrated when his symptoms would occur unexpectedly and was left lamenting why he even had asthma at all (Box 1, Quote 2). Children and parents also reported that the lack of control over asthma symptoms made them feel scared and worried (Box 1, Quotes 3 and 4).

##### Children’s machine creations

During the discussions of their machine creations, children expressed that they experienced negative emotions when their asthma symptoms worsened. Children described how they designed their machine creations to reduce their fear and make them happy and laugh. Safety was an important element of children’s creations to making them feel better. For some children, safety involved being removed from asthma triggers (Box 1, Quote 5). For other children, safety was achieved when their machines would notify parents or doctors about a flare-up of symptoms (Box 1, Quote 6).

Box 1 **Fear, sadness and frustration associated with asthma** **Quote 1**: “I feel playful because I’m not sick and I have lots of energy and I feel happy… I feel excited because it’s a better time instead of being all bad and have to sit down instead of playing.” (Child, focus group 7, ages 6–8 years)

**Quote 2**: “I just sometimes don’t even know why it happens and I’m like what the heck? What the heck? Why is this happening? Why me?” (Child, focus group 15, ages 6–8 years)

**Quote 3**: “When I was in hospital they got a lot of different things I didn’t know and so I just felt scared and frustrated because you keep coughing and it really hurts and you don’t know what to do about it.” (Child, focus group 10, ages 9–11 years)

**Quote 4**: “You feel a bit worried that you’re going to get so sick and it is pretty hard to control it even when you have your puffer so you get a bit scared that you are not going to get over it.” (Child, focus group 13, ages 6–8 years)

**Quotations from children’s machine creations**

**Quote 5**: “*Child*: That’s a magical bird and that is my human there … and it has asthma because it’s afraid of the [dusty] place and then the bird come and then take it away into the nature place.

*Interviewer:* And is that nature place not dusty?

*Child:* Yeah, not dusty.” (Child, machine creations group 1, ages 6–8 years)

**Quote 6**: “Child: The cheetah would get you up and run around with you and it can take you to the doctors really quickly.” (Child, machine creations group 13, ages 6–8 years)

#### Parental responsibility for proactively monitoring triggers, symptoms and medication

Children in the focus groups generally discussed their reactive management of asthma and what they do when their symptoms emerge. Parents, however, were more proactive and discussed preventative behaviours. Accordingly, parents were largely responsible for monitoring triggers, asthma symptoms and medication use. One challenge to children’s preventative medication usage, as described by parents, is that children often did not understand why they had to take their medication when they were not experiencing asthma symptoms (Box 2, Quote 1).

Children were generally aware of potential triggers for their asthma and the important preventative behaviours they needed to enact, including avoiding dusty places, avoiding allergens, keeping warm, and resting. However, children sometimes reported that they did not adhere to these preventative behaviours unless specifically reminded by their parents. This was true for children of all.

For parents, monitoring their child’s exposure to triggering situations was a challenge (Box 2, Quote 2).

Similarly, children of all ages were reliant on their parents to monitor symptoms and medication. While children were aware they needed to take their medication, they were generally unaware about the specifics such as dosage and frequency of medications (Box 2, Quote 3).

During the focus groups, parents and children indicated that children were not yet at a maturity level to be responsible for their own asthma and so parents inhabited this role. Several families indicated that older children were in the process of taking more responsibility for their asthma, particularly for when the child was staying with friends. Yet these children were still reliant on their parents to monitor their asthma at home (Box 2, Quote 4).

Parents reported various behaviours to monitor their child’s asthma, including mentally tracking symptoms and medication use, writing down symptoms and medication use, and checking the weather forecast to prepare for an asthma attack (Box 2, Quote 5 and 6). Parents suggested that apps could help them to track their child’s symptoms and medications. Parents emphasised the shared use of apps with their children, and that the purpose of apps should be to better facilitate information sharing between parents and children. Parents also suggested that apps could help their children gain the skills to slowly become more independent in their asthma self-management.

Parents were solely responsible for deciding whether their child’s symptoms were severe enough to warrant seeing a GP or to go to the hospital. Parents typically found this a difficult task and commonly reported feeling unsure about when to escalate their child’s care (Box 2, Quote 7).

##### Children’s machine creations

Children explained that their machines creations would sense when they needed their medication and dispense the appropriate dosage (Box 2, Quote 8). In this sense, children created machines which would take over the responsibility of monitoring their symptoms and medication use. Important to the children, the machine would make this process fun and enjoyable (Box 2, Quote 9).

Box 2 Parental responsibility for monitoring triggers, symptoms and medication **Quote 1:** “When they don’t have asthma it’s like, ‘Why am I doing this?’ And that’s what [my daughter] said the last few days, ‘I don’t feel wheezy.’ And it’s like, ‘We’ve talk about it.’…she knows but at this age it’s, to have to, it’s like ‘take medicine when you feel okay’.

*Q:* It’s tricky.

*F:* It’s hard.” (Mother, focus group 14, ages 9–11 years)

**Quote 2:** “*F*: I think controlling outside factors—I like to have the house clean, but my Mother-in-Law doesn’t so going to my Mother-in-Law’s house is stressful for me because I know she’s highly allergic to dust.” (Mother, focus group 1, ages 6–8 years).

**Quote 3:** “Q: Do you track or record how your asthma’s going at all?

*C:* No.

*Q:* Mum does that?

*C:* Yeah” (Child, focus group 12, ages 9–11 years)

**Quote 4:** “When she’s out with her friends … it’s up to her to take that responsibility to use [her inhaler].” (Mother, focus group 12, ages 9–11 years)

**Quote 5:** “I sometimes make notes on my phone of how often I am giving Ventolin so if we end up in emergency I can say I’ve done this this this and this.” (Mother, focus group 11, ages 6–8 years)

**Quote 6:** “It depends on how bad it is. Sometimes I do write [symptoms and medications] down so that when we go to the doctor I can tell him exactly what I’ve done when and what time and how bad it was. And sometimes I’ve even recorded his breathing for the doctor so the doctor can hear it.” (Mother, focus group 4, ages 6–8 years)

**Quote 7:** “In the past she’s just gone downhill very quickly, it’s hard because you don’t want to be every time she gets asthma go to the doctor or hospital or whatever, but at the same time knowing what’s happened previously it’s always in the back of your head what could happen … [do you] just stay home and monitor or do you go to the doctor early and try to nip it in the bud before it exacerbates?” (Mother, focus group 3, ages 6–8 years)

**Quotations from children’s machine creations**

**Quote 8:** “Child: I have a backpack that [delivers] the medicine…and never stop until they’re better.” (Child, machine creations group 9, ages 6–8 years)

**Quote 9:** “Child: If you push green [button] the icing cake comes around, if you push the yellow [button] then the magical Maltesers come out…

It would give you normal Ventolin but in a very fun way.” (Child, machine creations group 2, ages 9–11 years)

#### Managing asthma at school requires child communication about symptoms

Most parents reported that they became aware of flare-ups via observing symptoms in their child, including coughing, wheezing, shortness of breath and low energy. This, however, was sometimes a problem when children were at school and therefore parents could not monitor their child’s symptoms. Only one parent said that their child would actively and consistently communicate their asthma flare-up to their parents and teachers. For the rest of the families, children commonly waited to communicate their symptoms which one parent described as putting them on the “back foot” (Box 3, Quote 1).

A common issue was that school policy often dictated that inhalers were to be kept locked in the office. Parents were concerned that their child often waited too long to ask for it (Box 3, Quote 2).

Children rarely reported that their response to experiencing physical asthma symptoms was to tell a teacher. One parent speculated that her daughter was too embarrassed to tell a teacher about an asthma flare-up and did not want to miss out on school activities (Box 3, Quote 3).

Parents were largely responsible for communicating their children’s asthma needs to their school (Box 3, Quote 4). Parents described mixed experiences with schools and great variably in response to their child’s asthma between teachers and between schools (Box 3, Quote 4).

##### Children’s machine creations

Children described creating machines which would alert their parents when they were beginning to experience symptoms of an asthma flare-up while at school (Box 2, Quote 5). Children reported that their machine creations may alert their parents or doctors, but no children reported their machine would alert a teacher. This reflects parents’ speculation in the focus groups that children were reluctant to alert their teachers to an asthma flare-up.

Box 3 **Managing asthma at school requiring child communication about symptoms** **Quote 1:** “She doesn’t tell me, not until it’s like beyond - when she should have had a puffer 10 minutes ago she doesn’t say anything until it’s really bad and then you are on back foot trying to help.” (Mother, focus group 11, ages 6–8 years)

**Quote 2:** “I can tell when he’s got asthma and I give him some Ventolin – he won’t get ask for it. Once I picked him up from school and said ‘how was he?’, ‘Oh fine’ and I took him in a for a lung function and it was 76% and he was crook – terrible” (Mother, focus group 11, ages 6–8 years)

**Quote 3:** “I think it’s a bit of an embarrassment for [my daughter]. She won’t tell the teacher that her asthma’s bad because I think she thinks that she’s going to miss out on something so …I go in and tell [the teacher].” (Mother, focus group 12, ages 9–11)

**Quote 4:** “Some people get it, other people don’t, so, trying to get through to them to say, ‘look, he can look really, really well one minute but if he says he needs to go to the office, he needs to go. You are not to keep him in class and just see whether he’s trying to get out of class…it’s from teacher to teacher because I still think some people don’t get it.” (Mother focus group 15, ages 6–8 years)

**Quotations from children’s machine creations**

**Quote 5:** “The reason why those feathers are there, so then it would – so if I needed extreme help my Mum could - say I was on an excursion and she didn’t know where about’s … she didn’t know if they were on the beach or in the park or something, so then that will connect and it would fly to where I would be and then it would mean that I would be okay because my Mum will be with me.” (Child, machine creation group 1, ages 6–8 years)

## Discussion

This paper examined how school-aged children with asthma and their parents perceive and experience their asthma, and how they manage symptoms, prevention and medications both within and outside the home. We identified the impact asthma can have on children’s social and emotional wellbeing and highlighted how reliant school-aged children are on their parents to effectively manage their asthma. Our results have implications for children’s self-management of their asthma, particularly when they are at school and away from their parents.

Both parents and children of all ages indicated that they were not ready for children to independently self-manage their asthma medications and symptoms. Consistent with previous research, this was particularly the case during acute episodes, when parents were solely responsible for decision-making during acute episodes including whether to escalate care to a health professional^33^. However, families of older children reported that children were in the process of taking more responsibility for their asthma management, which was not reported for the younger children and is consistent with previous research^22^.

These findings echo previous research that found children with chronic illness may lack the cognitive, physical and psychosocial abilities to proactively and autonomously manage their medications^34^. According to Piaget’s Concrete Operational Stage of Cognitive Development, children aged approximately 7–11 years are logical thinkers and have not yet developed the ability to think abstractly and hypothetically^23^. These developmental profiles may explain why children in our study were reactive in managing their asthma, meaning they would begin management once their symptoms appeared. They struggled to understand why it was necessary to take their asthma medication when they were feeling well and were not experiencing active symptoms. Parents, however, used a proactive approach to their child’s asthma management to help prevent symptoms, and were largely responsible for monitoring triggers and medications. Clinical and self-management strategies should consider the complementary and shared roles children and their parents play in managing paediatric asthma, as well as different developmental trajectories across children’s age ranges. Services to assess the self-management knowledge and skills of children and their parents, and provide education and tools to help address identified gaps, are needed.

Despite being solely responsible for escalating their child’s care, parents commonly reported feeling unsure about when their child’s asthma symptoms warranted a visit to the doctor or hospital. These findings support training for parents to be able to detect the early signs of an asthma attack. Nurses and other health professionals are ideally placed to help educate parents about how to recognise worsening asthma symptoms^35–38^.

Some parents in our study wanted to empower their children to be part of the decision-making process for asthma management. Parents reported that interventions such as phone applications (apps) could have the potential to facilitate information sharing between parents and children and teach children important self-management skills. Apps can support asthma self-management and supplement existing clinical care through real-time monitoring, facilitating information sharing and addressing barriers to self-management (such as forgetting medication)^15,16,18^. Early evidence shows that apps that assist children to self-manage their asthma can result in improved outcomes such as reduced hospital admissions and missed school attendance^39^. Given the ubiquitousness of mobile usage generally in our sample, with 76% of children having used an app of some description, further studies outlining what works, particularly focussed on children’s developmental cognitive stages and reactive approach to management is warranted.

As well as the practical aspects of asthma management, children were also reliant on their parents for management of their asthma. Children reported negative emotional reactions to their asthma, including sadness, frustration and fear, in line with previous reporting^40^. Consistent with prior research, these reactions were particularly strong when their asthma prevented them from participating in activities with their peers^41,42^. Children were also scared by the uncontrollable nature of their symptoms, and in discussions about their machine creations they described the importance of seeking safety from their asthma symptoms by alerting their parents. In confirmation of past research, children with chronic illness soothe their fear by seeking comfort from their parents, particularly their mothers^43,44^. Peer interventions could be employed to alleviate some of the negative emotional responses children associate with their asthma, including sadness and frustration.

The challenges of managing children’s asthma at school were also another theme of our findings. Children’s avoidance of appearing different, and lack of understanding by teachers may cause children to avoid taking their medication at school. According to parents, schools would commonly keep children’s asthma inhalers locked in an office. Parents speculated that their children would avoid asking for their asthma medication when they were at school to avoid feeling different. Previous research also shows that children with asthma may ignore early symptoms of an asthma flare-up and avoid taking their asthma medication to prevent feeling “different” to their peers^45,46^. Children’s lack of access to their inhalers while at school has commonly been identified as a barrier to appropriate use of their inhaler^46–49^. Parents also reported that teachers were not always knowledgeable about asthma and their child’s asthma management plan, in agreement with previous research^45,46,51^ Building on existing literature^22^,^51–54^, future research may seek to more fully assess the perspectives of key stakeholders such as governing councils, school nurses and teachers. This research is needed to design and test solutions, particularly regarding access to children’s inhalers. Interventions to improve children’s self-management of their asthma at school may also aim to reduce the social stigma associated with using inhalers^50^.

A major strength of our study was the inclusion of both parent and child perspectives in the focus groups. Discrepancies have previously been reported between parent-reports and child-reports of child and family health^55,56^, so it is important to gain multiple perspectives. The use of the machine creations task allowed us to further facilitate discussion with the children about their perspectives on their asthma management. Some of these perspectives, such as children only alerting parents or doctors and never teachers during an asthma flare-up, were only derived from the discussions around the machine creations task. This may be perceived as a strength of the study as the machine creations gained children’s unique perspectives, or a weakness in that these perspectives were not triangulated with other data sources. Future exploration of the feasibility, acceptability and credibility of this data collection method may be warranted. As to limitations, families who were functioning well may have been more inclined to opt-in to our research. Indeed, 95% of the children in our study had a written asthma management plan, which is unlikely to be representative of the broader population. Given that family functioning is related to children’s asthma management^57,58^, this selection bias may have skewed our results and been amplified by the rate of participants who were lost to follow-up. Alternatively, our recruitment e-newsletter and physical notice boards mentioned the importance of good asthma management, and therefore parents who were in some way struggling with their child’s asthma management may have been more inclined to self-select into the study. In addition, no fathers participated in the focus groups, though this may reflect the often gendered approach to asthma care within families; mothers are typically more involved in their child’s asthma management than fathers^59^. Aboriginal and Torres Strait Islander families were underrepresented in the study, with only one participating, and information was not collected regarding other culturally and linguistically diverse (CaLD) backgrounds, meaning we are unable to observe how this may have influenced responses. We also did not gather data regarding socioeconomic status, family income or parent marital status. These factors have all previously been found to influence children’s asthma management, including structural barriers to implementing guideline-based care^57,60,61^.

This study shows that parents are largely responsible for the practical management of their school-aged children’s asthma at home, particularly during acute attacks. Services to educate parents on how to recognise symptoms of an attack could be beneficial. More research on phone apps that assist children’s self-management of asthma, or joint decision-making with parents, is warranted. These should be designed with children’s cognitive development stages in mind. Parents also provide the primary emotional support to children who may be self-conscious about their asthma and medication and appearing “different” to their peers. Outside of the home, schools can be a source of ongoing difficulties regarding managing their child’s asthma. School policies and teachers’ lack of understanding of asthma can exacerbate children’s reluctance to use their inhaler at school. Greater understanding of safe ways of children accessing their inhalers at schools is needed.

### Reporting summary

Further information on research design is available in the Nature Research Reporting Summary linked to this article.

## Supplementary information

Reporting Summary

## Data Availability

The focus groups guides are provided in Supplementary File C. The interview transcripts are not available due to ethics requirements and potential to identify participants.
