# Supplementary material for: Child and caregiver experiences and perceptions of asthma self-management
Source: NPJ Prim Care Respir Med. 2021 Sep 9;31:42. doi: 10.1038/s41533-021-00253-9 (PMC8429661; doi:10.1038/s41533-021-00253-9)
Supplement: Supplementary file 1 — Reporting Summary [file 41533_2021_253_MOESM1_ESM.pdf]

nature research

Corresponding author(s): Dr Lauren Kelada (l.kelada@unsw.edu.au)

Last updated by author(s): Mar 18, 2021

Reporting Summary

Nature Research wishes to improve the reproducibility of the work that we publish. This form provides structure for consistency and transparency in reporting. For further information on Nature Research policies, see our Editorial Policies and the Editorial Policy Checklist.

Statistics

For all statistical analyses, confirm that the following items are present in the figure legend, table legend, main text, or Methods section.

n/a	Confirmed	
☐	☒	The exact sample size (*n*) for each experimental group/condition, given as a discrete number and unit of measurement
☒	☐	A statement on whether measurements were taken from distinct samples or whether the same sample was measured repeatedly
☒	☐	The statistical test(s) used AND whether they are one- or two-sided<br>*Only common tests should be described solely by name; describe more complex techniques in the Methods section.*
☒	☐	A description of all covariates tested
☒	☐	A description of any assumptions or corrections, such as tests of normality and adjustment for multiple comparisons
☒	☐	A full description of the statistical parameters including central tendency (e.g. means) or other basic estimates (e.g. regression coefficient) AND variation (e.g. standard deviation) or associated estimates of uncertainty (e.g. confidence intervals)
☒	☐	For null hypothesis testing, the test statistic (e.g. *F*, *t*, *r*) with confidence intervals, effect sizes, degrees of freedom and *P* value noted<br>*Give P values as exact values whenever suitable.*
☒	☐	For Bayesian analysis, information on the choice of priors and Markov chain Monte Carlo settings
☒	☐	For hierarchical and complex designs, identification of the appropriate level for tests and full reporting of outcomes
☒	☐	Estimates of effect sizes (e.g. Cohen's *d*, Pearson's *r*), indicating how they were calculated

Our web collection on statistics for biologists contains articles on many of the points above.

Software and code

Policy information about availability of computer code

Data collection	No software was used
Data analysis	NVivo Version 12

For manuscripts utilizing custom algorithms or software that are central to the research but not yet described in published literature, software must be made available to editors and reviewers. We strongly encourage code deposition in a community repository (e.g. GitHub). See the Nature Research guidelines for submitting code & software for further information.

Data

Policy information about availability of data

All manuscripts must include a data availability statement. This statement should provide the following information, where applicable:

- Accession codes, unique identifiers, or web links for publicly available datasets
- A list of figures that have associated raw data
- A description of any restrictions on data availability

The focus groups guides are provided in supplementary file C. The interview transcripts are not available due to ethics requirements and potential to identify participants.

Field-specific reporting

Please select the one below that is the best fit for your research. If you are not sure, read the appropriate sections before making your selection.

☐ Life sciences ☒ Behavioural & social sciences ☐ Ecological, evolutionary & environmental sciences

For a reference copy of the document with all sections, see nature.com/documents/nr-reporting-summary-flat.pdf

Behavioural & social sciences study design

All studies must disclose on these points even when the disclosure is negative.

Study description	Qualitative: focus groups.
Research sample	We undertook 15 focus groups with 41 school-aged (6-11 years) children with asthma and 38 parents. No fathers participated in the focus groups and Aboriginal and Torres Strait Islander families were underrepresented.
Sampling strategy	Recruitment of participants (children and their parents) occurred in 2017 across four Australian states: New South Wales (NSW), Queensland (Qld), South Australia (SA) and Victoria (Vic) via e-newsletter circulations, and online or physical noticeboard advertisements through Asthma Australia (the peak national consumer body), schools, and Facebook. Field notes were made during and after each Focus Group, with researchers (CG, CM) reaching consensus on the main points and data saturation.
Data collection	Group discussions were semi-structured and led by a professional focus group facilitator and at least one member of the research team (CG and/or CM)(all female). In total, two professional facilitators were involved in the focus groups (one per session) and each held training in psychology and over seven years' experience in social and qualitative research and conducting focus groups with children. One research team member, the professional facilitator, and children with asthma and their caregivers, were present at each focus group. All sessions were audiotaped and transcribed verbatim for later analysis.
Timing	March to April, 2017.
Data exclusions	No data were excluded.
Non-participation	22 families who had expressed interest in the study but had not yet returned the consent form, were lost to follow-up prior to attending the focus groups. Reasons provided included illness, childcare arrangements (for other siblings) and other commitments (e.g., work).
Randomization	Participants were not allocated to experimental groups.

Reporting for specific materials, systems and methods

We require information from authors about some types of materials, experimental systems and methods used in many studies. Here, indicate whether each material, system or method listed is relevant to your study. If you are not sure if a list item applies to your research, read the appropriate section before selecting a response.

Materials & experimental systems

n/a	Involved in the study	
☒	☐	Antibodies
☒	☐	Eukaryotic cell lines
☒	☐	Palaeontology and archaeology
☒	☐	Animals and other organisms
☐	☒	Human research participants
☒	☐	Clinical data
☒	☐	Dual use research of concern

Methods

n/a	Involved in the study	
☒	☐	ChIP-seq
☒	☐	Flow cytometry
☒	☐	MRI-based neuroimaging

Human research participants

Policy information about studies involving human research participants

Population characteristics	See above.
Recruitment	Recruitment of participants (children and their parents) occurred in 2017 across four Australian states: New South Wales (NSW), Queensland (Qld), South Australia (SA) and Victoria (Vic) via e-newsletter circulations, and online or physical noticeboard advertisements through Asthma Australia (the peak national consumer body), schools, and Facebook. Field notes were made during and after each Focus Group, with researchers (CG, CM) reaching consensus on the main points and data saturation. 95% of the children in our study had a written asthma management plan, which is unlikely to be

representative of the broader population so families who were managing their child's asthma well may have been more likely to self-select into the study. Alternatively, our recruitment e-newsletter and physical notice boards mentioned the importance of good asthma management, and therefore parents who were in some way struggling with their child's asthma management may have been more inclined to self-select into the study.

Ethics oversight	University of New South Wales Human Research Ethics Committee (no: HC15733)

Note that full information on the approval of the study protocol must also be provided in the manuscript.
